# Microplastic Pollution Prevention: The Need for Robust Policy Interventions to Close the Loopholes in Current Waste Management Practices

**DOI:** 10.3390/ijerph20146434

**Published:** 2023-07-23

**Authors:** Hiroshan Hettiarachchi, Jay N. Meegoda

**Affiliations:** 1 Independent Researcher, 650 Trailwood Path, Unit D, Bloomfield Hills, MI 48301, USA; hiroshanh@gmail.com; 2 Department of Civil and Environmental Engineering, New Jersey Institute of Technology, University Heights, Newark, NJ 07102, USA

**Keywords:** plastic pollution, microplastics, prevention, remediation, policy interventions

## Abstract

Plastic materials that are less than 5 mm in size are defined as Microplastics (MPs). MPs that are intentionally produced are called primary MPs; however, the most abundant type in the environment consists of the remainder created by the fragmentation of large plastic debris through physical, chemical, and oxidative processes, which are called secondary MPs. Due to their abundance in the environment, poor degradability, toxicological properties, and negative impact on aquatic and terrestrial organisms, including humans, MP pollution has become a global environmental issue. Combatting MP pollution requires both remediation and preventive measures. Although remediation is a must, considering where the technology stands today, it may take long time to make it happen. Prevention, on the other hand, can be and should be done now. However, the effectiveness of preventive measures depends heavily on how well MP escape routes are researched and understood. In this research, we argue that such escape routes (rather, loopholes) exist not only due to mismanaged plastic waste, but also due to cracks in the current waste management systems. One known MP loophole is facilitated by wastewater treatment plants (WWTP). The inability of existing WWTP to retain finer MPs, which are finally released to water bodies together with the treated wastewater, along with the return of captured larger MPs back to landfills and their release into the environment through land applications, are a few examples. Organic waste composting and upcycling of waste incineration ash provide other MP escape pathways. In addition, it is important to understand that the plastics that are in current circulation (active use as well as idling) are responsible for producing MPs through regular wear and tear. Closing these loopholes may be best attempted through policy interventions.

## 1. Introduction

Plastic is becoming increasingly common in nature, not only due to its massive production rate but because of mismanagement of it as a material, especially when it becomes a waste product. The abundance of plastic waste in the environment and the disintegration of larger plastics into smaller pieces have led to the recent environmental issue that has been named microplastic pollution. This naming follows the size spectrum of these smaller plastics, which are predominantly in the micrometer range. As per the currently accepted definition, any piece of plastic smaller than 5 mm is a microplastic (MP) [1].

Although the upper bound is well defined, the lower bound of this spectrum remains under debate. Smaller pieces of plastic in the nanometer range (plastics smaller than 1 micrometer) are usually called nanoplastics due to their distinctively different behavior, although a proper definition for nanoplastics is yet to be found [2]. For the purpose of this discussion, in this manuscript we consistently use the general term MP to indicate all plastics smaller than 5 mm.

MPs can be made up of many different thermoplastic polymers plastic types, such as– polyethylene terephthalate (PET), polyethylene (PE), polypropylene (PP), polystyrene (PS), and polyvinyl chloride (PVC), and can be of any color and can come in many different shapes, such as fragments, fibers, pellets, foams, films, and more. The discussion here does not include elastomers and thermosetting plastics, as they do not undergo depolymerization, or bio-based and biodegradable plastics. Most MPs result from commonly used and post-consumer plastic waste. Irrespective of type, color or shape, MPs are categorized as primary or secondary based on their origin. Plastics that are manufactured for commercial use and small enough to be in the above-defined MP size range are defined as primary MPs. These plastics are typically used in facial cleansers and cosmetics, and sometimes in medicine as vectors for drugs [3]. Secondary MPs are fragments created from the breakdown of larger plastic pieces or macroplastics [4] such as plastic litter, fiber from synthetic textiles, and tires. This fragmentation is caused by cumulative effects of physical, biological, and chemical processes over time [5]. Exposure to sunlight over prolonged periods is one of the main contributors of secondary MPs. Weathering is the primary process for plastic degradation [6,7]. Known as photo-fragmentation, ultraviolet (UV) radiation from the sun oxidizes the plastic polymer, resulting in chain scission [8]. The thermoplastics are often subjected to so-called UV radiation, where sunlight leads to the shortening of macromolecular chains and the reduction of the molecular weight of thermoplastic polymers; this affects their macroscopic behavior and leads to deterioration of their mechanical properties. Photo-fragmentation causes the material to become increasingly brittle until it reaches mechanical failure. The resulting loss of structural integrity makes these plastics more susceptible to fragmentation due to abrasion, wave action, and turbulence, resulting in the production of more and more secondary MPs. This progression occurs continuously, with each piece breaking down into smaller and smaller pieces.

The gravest issue surrounding MP pollution is its toxicity to the environment and to living beings. Therefore, it is important to understand the impacts of MPs on the environment, humans, animals, etc. Typical additives to plastics include perfluorinated chemicals, phthalates, bisphenols, polybrominated diphenyl ethers, and nonylphenols, for which toxicity to both humans and the ecosystem are well established. In an aqueous environment, plastic floats freely and can carry items from one place to another, acting as a potential carrier of invasive species in marine environments [9]. MPs are toxic to marine life, and for similar reasons are toxic to humans. In terrestrial environments, MPs can affect soil fertility and soil organisms [10]. For example, MPs can affect the development and mortality of earthworms [11]. It should be noted that chemicals are often deliberately added during the production of plastic to enhance performance attributes such as strength, flexibility, stiffness, and various other desirable properties. More than 10,000 chemicals are used during plastic manufacturing, and at least 2000 of those are known for having health impacts on humans as well as on the environment [12]. It is well known that both the plastics themselves and these additives can be toxic and disruptive to the proper functioning of the human body. For example, Bisphenol-A (or BPA) and phthalates prevalent in plastic manufacturing are associated with negative health impacts such as infertility, cardiovascular disease, and cancer [13].

The information presented above justifies why combatting MP pollution is a necessity. Combatting such pollution essentially involves remediation of polluted environment as well as application of preventive measures to stop further spreading. MP remediation is an emerging field. The current understanding of the fate of MPs once they enter water, soil, and living matter, as well as how they affect other nutrients or pollute their respective environments, is not yet fully developed. It may take quite some time before protocols for detecting and measuring MP concentrations, especially at the nanoscale, can be made implementable. In the meantime, preventive options can play a key role in slowing the spread of MP pollution. There are many preventive actions that can be promoted/executed, such as to eliminate the misuse of plastics and mismanagement of plastic waste. In this context, the objective of this paper is to investigate both apparent and hidden MP escape routes in current material/waste management practices and how such loopholes can be closed with the help of appropriate policy interventions.

## 2. Background

The MPs that impact living beings, including humans, come from the environment. First, MPs pollute the environment, then they enter humans/animals through food, water, or the plastic products they associate with. A brief discussion of the current understanding of the major entry points of MPs into the environment, their health impacts, and different methods for combatting MP pollution is presented in the following subsections.

### 2.1. From the Source into the Environment

The release pathways of MPs into the environment are abundant and varied. One of the main known points of entry into the environment is Wastewater Treatment Plants (WWTPs) [14]. Residential, commercial, and industrial wastewater already contains MP due to the presence of items such as cosmetics, hygiene products, wet wipes, facial scrubs, cigarette butts, and cleaning products. WWTPs treat wastewater in three stages: primary, secondary, and tertiary. Primary treatment attempts to physically settle insoluble solids such as MPs through a variety of methods, including screening, grit removal, and oil and grease removal. Secondary treatment is primarily concerned with biological aspects of wastewater and secondary settling. During the primary and secondary treatment processes of WWTPs, approximately 88% of MPs are separated into the wastewater sludge [15,16,17,18]. All advanced WWTPs are equipped with tertiary treatment, during which the wastewater is subjected to further filtration followed by additional disinfecting treatment. Although these three processes (primary, secondary, and tertiary treatment) are able to remove about 90% of MPs from wastewater, they are not effective in removing MP particles smaller than 250 μm [14]. In addition, the MPs retained together with sewage sludge during the three stages of treatment, can re-enter terrestrial ecosystems when/if sewage sludge is used for land applications (defined as biosolids). MPs that enter terrestrial ecosystems through land-applied biosolids can then re-enter the aquatic environment via stormwater runoff [19].

Food waste is another important route by which MP enter aquatic environments. MPs have been detected in a wide variety of foods, including shellfish, rice, fruit, and vegetables. A study by Dessi [20] found that plastic contamination in pre-cooked instant rice was four times greater than in uncooked rice. The plastic packing/containers that are typically used with food for immersing in hot water, microwaving, and refrigeration have been shown to produce MPs. Styrofoam, a material commonly used for making disposable food containers, is known for releasing MPs when subjected to high temperatures [21,22]. Moreover, the way in which organic waste is collected can lead to additional MP contamination. In many municipalities and countries food waste is collected in plastic bags, and unsorted plastic waste such as disposable cutlery and plastic packaging inevitably becomes mixed up with food waste during disposal. This mixed waste usually ends up either at a landfill or a composting facility. The plastics that arrive at a landfill with food waste degrade into MPs over time. Although macroplastics may be screened out during processing at a composting facility, this is not the case for MPs. During composting and subsequent farming these plastics become further degraded. MPs in the soil may then be taken up by plants, leading to human or animal consumption [23].

The negative connotation attached to the word “pollution” usually suggests that it is due to mismanagement of plastic materials/waste. However, in the case of MPs the pollution occurs not only due to mismanagement but also due to wear and tear of the items made of plastics that we use in our regular day-to-day activities. Sources of MPs from daily activities can range from consumer products (e.g., synthetic textiles, single-use plastics, personal care products, and packaging) to transportation (e.g., erosion of synthetic rubber tires) to agriculture (e.g., plastic mulch, biosolids) to recreation (e.g., artificial turf).

### 2.2. From the Environment into the Human Body

Inhalation, contact, and consumption are the three intakes through which chemicals enter the human body [24]. Of the three, the main intake is consumption, i.e., food and drinking water [25,26]. The World Health Organization (WHO) identifies the key sources of MP pollution in fresh water sources as terrestrial stormwater runoff and wastewater effluent [27]. Because WWTPs are unable to remove finer MPs, when the treated wastewater is discharged to the environment these finer MPs follow and enter water bodies, eventually becoming present in the water cycle and entering drinking water [27,28]. Several previous research studies have described the damaging impact of MPs in drinking water [29,30,31,32]. A recent study conducted on water samples from 259 different examples of bottled water sold in several countries revealed that about 93% contained synthetic polymer particles [33]. It is believed that MP < 10 μm are mostly associated with drinking water sold in plastic bottles [34].

As a result of plastic pollution of aquatic resources, fish and other aquatic animals have become contaminated (or rather poisoned) with MPs. Zebrafish have specifically demonstrated great aversion to MPs, which cause excessive oxidative stress within the organism [35]. Evidence shows that MP pollution specifically can lead to intestinal inflammation, decreased intestinal microbiota diversity, immune system disruption, and decreased mucus secretion in marine animals. These findings have been found to apply to mammals as well, including humans [36]. MPs present in fish can enter the human body directly through consumption of fish or indirectly through fishmeal, which is commonly found in animal feed [25].

After being ingested or inhaled, MPs linger in the intestinal tract; those pieces that are 150 μm or larger in their greatest dimension stay bound to intestinal mucus. MPs that are less than 150 μm in their greatest dimension can be diffused into the bloodstream, where they can potentially disrupt the immune response [36]. It has been shown that nanoscale MPs can penetrate even further into organs via the blood due to their miniscule size [37]. Human breastmilk samples collected from 34 women and analyzed in a pilot single-center observational study using Micro-Raman spectroscopy revealed that 26 contaminated with MPs [37]. Another study reported that 50 nm polystyrene particles could pass through the blood–brain barrier [38]. In addition, it has been proven that inhalation can act as a route of exposure for environmental MPs [39].

Lingering MPs in the gut can lead to intestinal oxidative damage, where there is an imbalance of the production and detoxification of reactive oxygen species [39]. If left un-checked, this can lead to damage of cell structures such as DNA, proteins, and lipids [39]. This in turn can lead to serious inflammatory bowel disease and colon cancer. Other impacts include issues with the permeability of the gut lining, which can result in MPs entering the bloodstream and other organs [39]. MPs can additionally disrupt the intestinal immune system, which is important to the ability of the intestine to handle threats, toxins, and food antigens [40]. As mentioned earlier, toxic additives that are usually present in MPs can absorb contaminants and pathogens, both of which may be toxic to the gut. Specifically, MPs can adsorb persistent organic pollutants, including organic molecules that are resistant to degradation and toxic to human health [41]. In addition, MPs inside the gut lining can host colonies of unwanted bacteria on their surface [41]. 

### 2.3. Combatting MPs: Remediation Versus Prevention

As with any other prolonged environmental pollution issue, MP pollution should be delt with through a combination of remediation and preventive measures. While remediation helps to clean up the environmental damage that pollution has already caused, preventive measures attempt to curtail further pollution. Remediation and prevention are both essential aspects in combatting a pollution issue, as the function served by one cannot be replaced with the other.

While remediation of MP-infested terrestrial or aquatic environments is a must, there are many challenges that prevent its immediate implementation. As mentioned before, MPs vary in size from a few millimeters to pieces that are invisible to the naked eye. Managing visible municipal solid waste is already challenging enough, and it is natural to see slow progress on managing MPs which are partly invisible. There are no established techniques to clean up MP pollution in most scenarios, such as in the ocean or the soil. Because this is an emerging scientific discipline, the technology needed for detecting MP-polluted environments, isolating/removing MPs, and treating MP pollution remains hard to come by. Even the handful of methods that have been identified/proposed are not necessarily cost-effective.

Bioremediation has been proposed to reduce microplastic-contaminated environments, particularly soil [42]. Bioremediation is the use of microorganisms to break down MPs via hydrolysis. Soil is a habitat for a wide range of microorganisms such as bacteria or fungi which can break down plastics by secreting enzymes that perform hydrolysis on MPs [43]. These are eventually turned into monomers, which the microorganisms can digest into carbon dioxide and water. Bioretention cells, a type of treatment for runoff water, have been proposed as an effective method of removing MPs from urban stormwater [44]. The MPs found in urban stormwater ae most likely secondary MPs resulting from abrasion of larger MPs. Bioretention cells are depressions in the ground where stormwater runoff is collected and treated. It has been shown that a median 84% decrease in MPs (106–5000 μm) can be achieved through bioretention [44].

On the other hand, preventive measures to control MP pollution have been gaining traction thanks to activities to raise awareness. Source reduction is perhaps the best method available to curtail further spread of MP pollution. This essentially requires further efforts in reducing plastic usage, increasing recycling, and engaging in public outreach to eliminate littering. In order to achieve a reduction in plastic usage, countries are currently introducing regulations on most commonly used single-use plastic items, although the same effort has not yet been seen with regard to curtailing the use of plastics in packaging materials [45]. Eliminating overuse of compost and fertilizer could help reduce soil contamination with MPs as well [42]. Use of plastics that are biodegradable, perhaps such as those made from starch, cellulose, or similar polymers, has been proposed and employed. However, biodegradable plastics involve other sustainability concerns, as the production process consumes a lot of water and land and most of these polymers are not recyclable after the first use.

## 3. Status of MP Pollution Preventive Actions around the Globe

Many countries and international organizations have been reacting to MP issues through new policies, laws, and regulations in various capacities. Policy interventions can play a very important role in enhancing MP preventive measures. Contemporary developments in MP preventive measures are briefly discussed below.

### 3.1. Recent Country-Specific Examples

A few countries have recognized microbeads in hygienic/cleansing products as a major source of MPs and taken steps to ban them. Such bans are a good example of ways to stop MP pollution at the source. For example, the United States Congress introduced the Microbead-Free Waters Act in 2015 to prohibit manufacturing, packaging, and distribution of rinse-off cosmetics that contain plastic microbeads [45]. A rinse-off cosmetic is any cosmetic product that is intended to be removed after use, such as shampoo, shaving cream, and toothpaste. The Microbead-Free Waters Act 2015 describes a “plastic microbead” as any plastic particle of less than 5 mm in size, agreeing with the current definition. The main objective of this law is to prevent rinse-off cosmetic products containing any intentionally added primary MPs [46]. In addition, MP pollution has captured the attention of the Draft National Strategy to Prevent Plastic Pollution, which was unveiled by the United States Environmental Protection Agency (USEPA) in April 2023 [47]. This draft strategy has three goals and preventing MPs/nanoplastics from entering waterways is specifically a part of the third goal.

Canada proposed a new plastic waste management system in 2020. It is a comprehensive management system with an ultimate goal of reducing the amount of macroplastics as well as MP discharge into the environment [48]. Addressing the sources of MPs such as microbeads in personal care products were among the main objectives of this law [48]. The Canadian government performed surveys until the end of 2021 to obtain public input on this new plastic waste management system. In addition, Canada started prohibiting the manufacture and importation of all toiletries with microbeads in 2018.

In 2020, Australia phased out microbeads in rinse-off cosmetics and personal care products. The Australian government is aiming to introduce regulations on plastic waste and phasing-out of PVC (polyvinyl chloride) plastic labels. The Australian National Plastics Plan, which was launched in 2021, outlines a plan to eventually phase out plastics from the Australian economy altogether [49]. It includes phasing out problematic plastics, making oceans and beaches free of plastic, and creating legislation to ensure waste responsibility, invest in recycling capability, invest in research, and continue to support recycling.

In addition to the above-mentioned specific examples, France, Italy, the Republic of Korea, New Zealand, and Sweden have all introduced national laws banning the use, sale, and/or manufacture of microbeads in personal care products, as per a UNEP report published in 2018 [45]. The same report stated that Belgium, Brazil, India, and Ireland were in the process of introducing laws banning microbeads.

**Figure 1:**
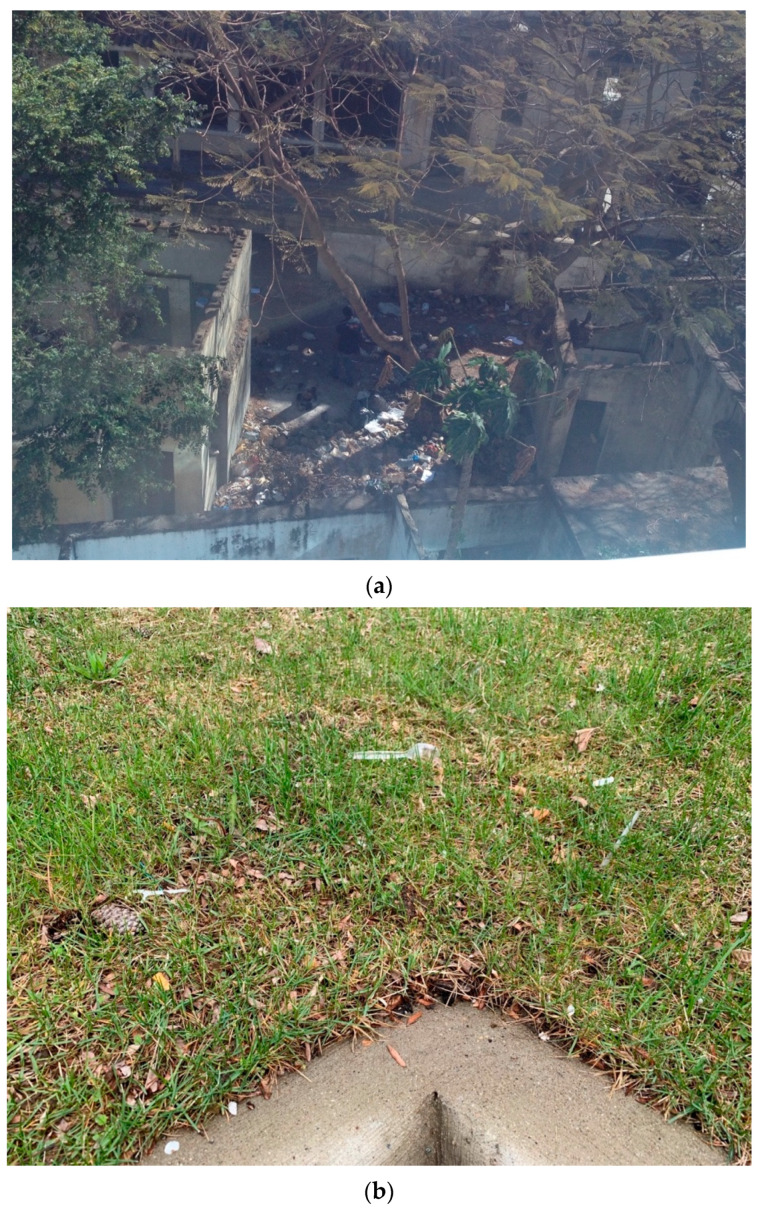
Mismanaged plastic waste is an issue common to developing as well as developed countries: (**a**) near a tourist hotel in Maputo, Mozambique in 2014 and (**b**) next to a parking lot in Michigan, USA in 2023.

Banning single-use plastic items is another preventive action reported from around the world. Banning single-use plastic items can essentially cut down a sizable fraction of MPs that easily slip through the waste collection system due to their small size and/or mismanagement (see Figure 1). The increasing public engagement and political commitment on this topic is displayed by the fact that more than sixty countries have already taken measures (i.e., heavy taxation or banning) to control single-use plastics and/or plastic bags [50,51]. An interesting trend of large cities being proactive to enforce such bans is emerging, possibly because national-level bans take more time to materialize. For example, although there is no national-level ban in the US yet, the UN Environment Program [50] indicated that ten US cities are already imposing their own bans. These ten cities are among forty other such cities from the around the world.

The potential for wastewater technologies to capture particles before they reach surface waters has begun to attract attention as well, although not yet at a sufficient scale [16]. For example, United Kingdom Water Industry Research (UKWIR) recently recommended intervention control for the wastewater industry [52]. This includes changes to labeling, extended producer responsibility, bans, grants and subsidies for reusables, and public infrastructure changes such as providing waste bins for disposal.

### 3.2. Global Efforts

The 2030 Agenda put forward by the United Nations (UN) and ratified by 193 member states did not capture MP pollution among any of the seventeen Sustainable Development Goals (SDGs) defined in 2015, at least not directly. If there is anything within the SDGs that touches upon the importance of combatting MP pollution, it is Indicator 14.1.1 in SDG 14 (life below water), which briefly talks about floating plastic debris in the oceans [53]. However, the United Nations Environment Program (abbreviated as UNEP or UN Environment), which is the key environmental program within the UN, has launched a few programs/campaigns to combat MP pollution, especially in recent years. While some of these specifically target MP pollution, in others combatting MP is incorporated into the umbrella topic of plastic pollution.

The United Nations Environment Assembly (UNEA) is what the UN defines as the world’s highest-level decision-making body on the environment and has passed four resolutions on marine litter since 2014 [54]. The UNEA is one of the few international organizations that recognize the MP issue, noting it as a threat to the environment as early as 2014. They have further recognized the lack of any existing global governance framework that can effectively deal with marine litter and microplastics issues. During the third assembly of the UNEA that took place in 2017, an expert group was established to look for solutions. In the fifth assembly, convened in 2022, the UNEA unanimously agreed that developing a legally binding treaty to combat plastic pollution would be a commendable step [55]. This is undoubtedly the most important development related to MP pollution in its entire history, and perhaps the most important and ambitious global-level environmental action since the Montreal Protocol in 1989. The UNEA has convened an intergovernmental negotiating committee in the hope of completing this work by the end of 2024 [56].

Another important step taken by the UNEA was to establish the UN Alliance for Sustainable Fashion during their fourth assembly in 2019. As per UNEP reports [57], they have teamed up with the other UN agencies in this alliance to actively campaign for government-level action to transition towards a sustainable textile industry with minimal release of MPs to the environment. This effort is particularly important considering that about 60% of clothing material is comprised of polyester, acrylic, and nylon, accounting for 9% of MPs received by the oceans each year [58]. Although there is an active awareness campaign, it is unclear whether anything substantial has been accomplished at the governmental level yet.

The European Commission (EC), which is the executive arm of the 27-member European Union (EU), adopted a broad Circular Economy Action Plan in 2020 aiming to address the presence of MPs in the environment [59]. This ambitious plan includes restricting intentionally added microplastics such as microbeads as well as developing labelling, standardization, certification, and regulatory measures on the unintentional release of MP. In the following year (2021), the EU set a target of reducing MPs released into the environment by 30%, and it is one of the key targets to be achieved by 2030 under EU’s Zero Pollution Action Plan for air, water, and soil. With the unveiling of the EU Strategy for Circular and Sustainable Textiles in 2022, the EC announced their intention to address MP pollution caused by synthetic fibers. Later in the same year, the EC proposed measures to limit plastic pollution from vehicle tires [58].

## 4. Preventive Measures Falling through the Cracks in Waste Management

While it is essential to employ the currently available remediation/prevention options in combatting MP pollution, it is important to understand their limitations as well. Their effectiveness in combatting MP pollution depends on how well and how fast these limitations are understood. This aspect is specifically important with regard to preventive measures. Even if the environment is somehow fully cleaned up through remediation techniques, the effort will be in vain if MPs keep returning to the environment due to the ineffectiveness of current preventive measures.

There is a common belief that better solid/liquid waste management practices will lead to better pollution preventive measures. Unfortunately, this is only partially true concerning MPs. The main reason for this issue is that unlike the other waste types, MPs are not responsive to most of the waste management methods currently in use, mainly due to their size and where/how they originate. In short, MPs easily fall through the cracks in existing waste management practices, including practices that involve advanced technology and that work for other typical solid/liquid wastes.

### 4.1. Plastic Waste Statistics

Global plastic production was about 2 million tonnes in 1950 and has soared ever since, surpassing 200 million tonnes/year just within 50 years [59]. Annual production reached 460 million tonnes in 2019, which is more than double the amount produced in the year 2000 [58]. With this growth in production/usage, global plastic waste generation has increased exponentially. Per the estimates made by Geyer et al. [60], approximately 8300 million tonnes of virgin plastics in total had been produced and 6300 million tonnes of plastic waste had been generated on a global scale as of 2015. The same study revealed that out of this global total, about 9% had been recycled, 12% incinerated, and the remaining 79% either accumulated in landfills or in the natural environment.

Although it is not easy to separate the global total of plastic waste accumulated in the environment from what is buried in landfills for the entire history of plastics production since the 1950s, inferences can be made by looking at the data available for recent years. For example, per reports of the Organization for Economic Co-operation and Development (OECD), the global total of plastic waste mismanaged or uncollected in the year 2019 was 22%, while about half (49%) was landfilled, 19% incinerated, and 9% recycled [61]. These global average percentages are compared in Table 1 with the averages for OECD countries and non-OECD countries.

It is evident from Table 1 that there is a drastic difference in plastic waste mismanagement between OECD countries and non-OECD countries, linking the MP issue to developmental issues. While plastic mismanagement within the OECD is limited to 6%, the same for non-OECD countries is a whopping 39%. Although not a binding definition, the usual understanding is that the OECD is comprised mostly of developed countries, meaning that the much higher non-OECD average indirectly represents the aggregate mismanagement that mainly happens in developing countries. Although these numbers in Table 1 only represent plastic waste, this mismanagement in developing countries is not limited to the plastics and extends to all types of waste. A report by the World Bank in 2012 revealed that about 30–60% of all waste in developing countries is not subject to any collection [62]. The same report pointed at lack of source separation capabilities as a main reason for the poor rates of recycling and recovery observed in many developing countries [62].

### 4.2. MP Escape Routes in Waste Management

**Figure 2:**
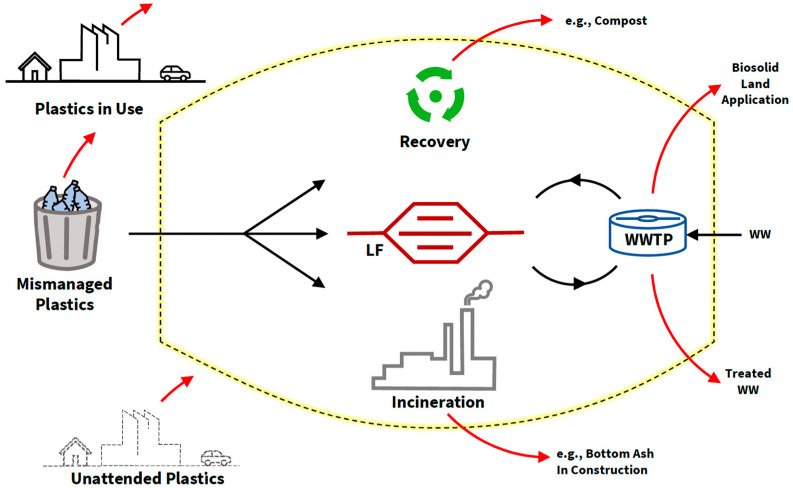
MP pollution falling through the cracks in current liquid/solid waste management practices. Key: LF—Landfill, WW—Wastewater, WWTP—Wastewater treatment plant, Dotted line—waste management system, Black arrow—waste flow, Red arrow—MP escape route.

Thinking along the traditional lines of waste management leads our focus only on to the mismanaged portion of plastic waste (e.g., 22% in 2019), as this may be the part that poses the biggest threat to the environment, including release of MPs. This mainstream thinking is evident in most efforts to combat MP pollution, which usually promote better waste management practices as a solution. Most MP awareness campaigns, and even the few new regulatory actions taken to date (as discussed before), indirectly assume that increased waste collection and recycling will be proportionately translated into prevention of MP pollution. The truth, however, is that the release of MPs into the environment is not limited to this unmanaged portion of plastic waste; in fact, it can even happen during the recycling, incineration, and landfilling processes (see Figure 2).

Where proper plastic waste management exists, one may conveniently assume landfills to be the final safe resting grounds for MPs. However, landfills offer MPs an escape route. When waste lies in a landfill for a long time, MPs have the ability to mix with the leachate, which is usually collected and sent to a WWTP for treatment. As discussed before, the current technologies employed at WWTPs are able to screen out about 90% of MPs, while the rest escape to the environment together with treated wastewater. The important point here is the pathway of sludge, including the MPs retained in it. Although WWTPs are able to retain a high percentage of MPs in sludge, currently there is no mandate or technology to isolate MPs from the rest of the materials in sludge. The sludge is generally landfilled, incinerated, or applied to land as biosolids. As previously discussed, land-applied biosolids are an easy escape route for MPs to return to the natural environment. If this sludge is incinerated, it is possible for unburned MPs in the bottom ash to escape into the environment, as explained further in the next paragraph. If landfilled, MPs in the sludge will follow the same cycle, being recirculated between WWTPs and sanitary landfills while making additional finer MPs in the meantime (see Figure 2). In this sense, between the landfill and the WWTP MPs find an infinite loop with a number of inbuilt escape routes.

Although incineration is widely accepted as a process that can permanently eliminate plastic waste, unburned material exists in the bottom ash, which is the solid residue from incinerators. A recent study revealed the possibility of 1 tonne of plastic waste producing 360 to 102,000 microplastic particles after incineration [63]. The main two options for disposing of bottom ash are sending it to a landfill for final disposal or using it as raw material in other manufacturing/construction processes, such as bricks, concrete, soil improvement, and pavement. Both options essentially create additional loopholes for MPs to avoid the existing waste management process.

Plastic recycling contributes to MP pollution as well, at least indirectly. When plastics are recycled, while a certain (minor) fraction of discarded material and the waste from the recycling process is retained within the waste management cycle, a majority of the material is re-introduced to industry for manufacturing new materials/goods. Per estimations by Geyer et al. [60], out of the 6300 million tonnes of plastic waste that the world has produced as of 2015, about 500 million tonnes have re-entered the market through recycling. Although recycling is a positive step, it must be noted that the plastics that have received a new life (from waste back into usage by society) will continue to release more MPs in this second life.

**Figure 3:**
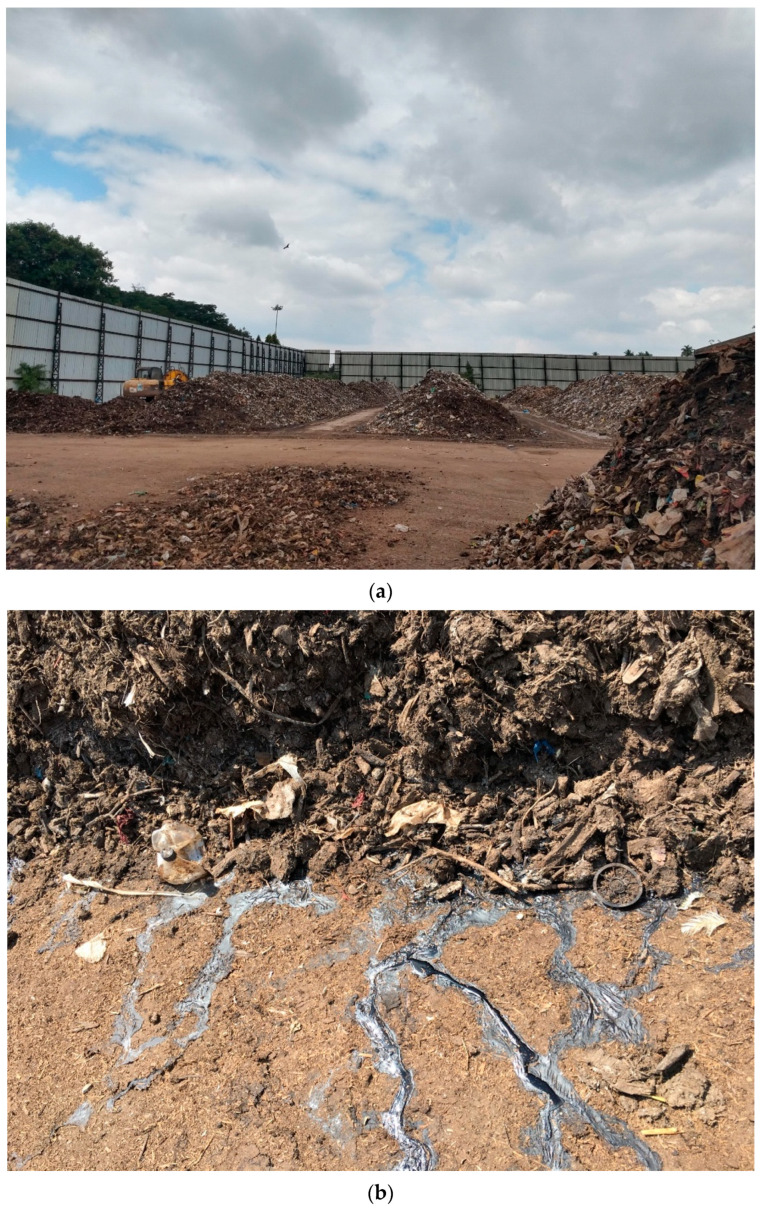
Plastic pollution in compost is an issue common to both developing and developed countries: (**a**) a composting plant near Mysuru, India in 2019 (picture credit: Namrata Mhaddolkar) and (**b**) a composting plant near Ramat Yishai, Israel in 2018.

In addition to the two material recovery processes discussed above (plastic recycling and bottom ash upcycling), organic waste composting offers MPs another escape route [64]. Organic waste composting is certainly a sustainable way to recycle nutrients; however, plastic pieces are known to cause quality control issues in compost made of organic waste (Figure 3). Although larger pieces of plastic can be screened out, currently there is no process to control/eliminate much smaller ones.

**Figure 4:**
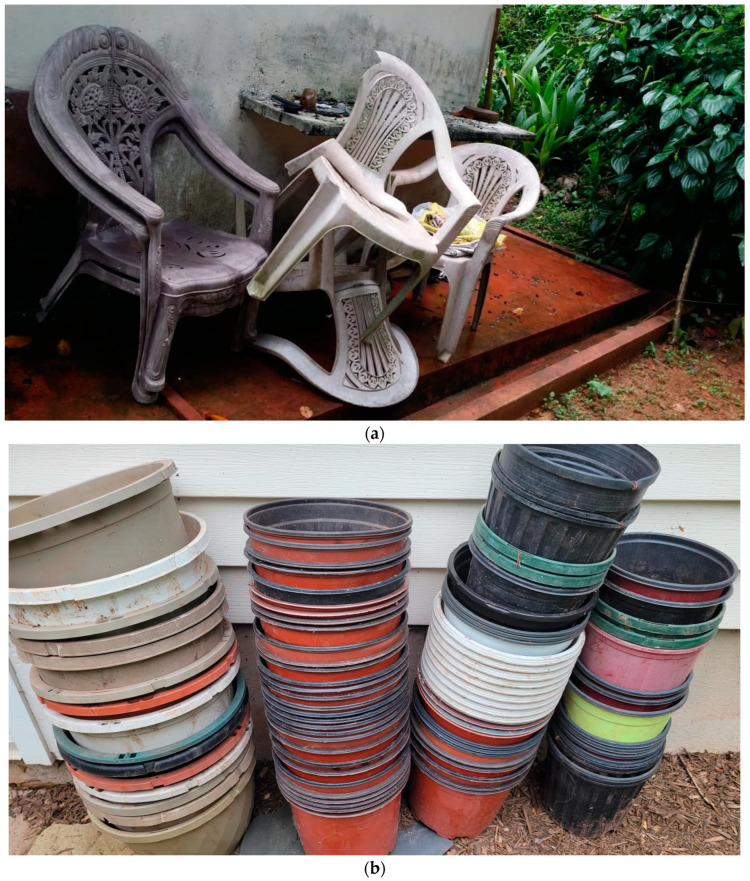
An example of (**a**) a pile of unattended plastics in a backyard near Kalutara, Sri Lanka, a developing country (photo credit: Mahesh Hettiarachchi) and (**b**) another in Hillsborough, NJ, USA, a developed nation.

In fact, release of MPs into the environment is not limited to waste plastics and may happens outside the traditionally recognized waste management/treatment enclosure (which is indicated by the dotted line in Figure 2). Literally, it happens everywhere, though it may not be visible because of the traditional narrative used in defining materials (in this case plastic) as waste or not. Those plastics that are already identified as material that is not useful anymore, such as used plastic utensils, broken plastic furniture, old synthetic fabrics, etc., clearly become unwanted material and eventually are labeled as waste and documented as such. Following the traditional waste management thinking, the global total of plastic waste discussed earlier is only about this portion that is labelled and/or documented as waste, of which some enters the waste management process, and the rest sits outside of it as mismanaged waste (see Figure 1 and Figure 2). However, what about the vast amounts of plastics that is not labelled as waste? This obviously includes the large number of plastics in use and the rest that are not currently in effective use yet not thrown out as waste either, i.e., unattended plastics (see Figure 2 and Figure 4). These plastics continue to release MPs through regular wear and tear. Returning to the numbers published by Geyer et al. [60], the difference between the global total produced by 2015 and the amount that has been labelled as waste is 2000 million tonnes.

The plastic waste that has been “saved” through recycling is close to another 500 million tonnes. These 2500 million tonnes are the plastics that remain in the possession of our society in both forms, that is, in active use as well as unattended plastics. What this means, in other words, is that there is a very large amount of plastic material among us which may be contributing to MP pollution through normal wear and tear.

## 5. The Need for Robust Policy Interventions

Campaigns against plastic/MP pollution have clearly gained momentum, especially in the past few years. Asking to enhance plastic waste management capacities through actions such as recycling can be seen as a popular demand. Although enhanced recycling can help to address the larger issue of plastic pollution, there is little it can do to curtail MP pollution. Based on the information presented in this review, there is compelling evidence to say that the waste management practices currently in use have holes in them that allow MPs to escape (Figure 2). When the existing waste management system is not sensitive to the scale and the extent of MP pollution, it is futile to expect better results from the same. The results will not be any better just because we are trying harder. Therefore, it is inevitable that the current waste management practices need to be tweaked somehow to close down the MP escape routes in them.

This is where new policy interventions are needed. Whenever there is compelling evidence of an imminent threat to the society or the environment, new thinking needs be formalized into new policies or existing policies need to be amended accordingly to assist in the process of implementing solutions. There are a few excellent historical examples for such policy interventions that have made revolutionary changes in mainstream thinking and actions. The Resource Conservation and Recovery Act (RCRA), enacted by the United States Congress in 1976, is one such country-specific example [65]. This was done in response to the growing volume of municipal and industrial waste in the US. In fact, the RCRA was an amendment of the earlier Solid Waste Disposal Act of 1965. It is interesting to note how many other countries were positively influenced by the RCRA; many other countries introduced similar policies in the years to follow and mimicked aspects of RCRA in their own policies. The decision to ban chlorofluorocarbons (CFCs) is another example, and one that portrayed excellent international cooperation. CFCs used to be a chemical important to several industries (such as refrigerants, blowing agents, aerosol propellants, etc.); however, when the evidence that CFCs were damaging the ozone layer emerged, pretty much the whole world rushed to support banning these chemicals through a treaty called the Montreal Protocol and its subsequent amendments. This happened within a in a very short period from 1987–1990. It is imperative that MP pollution prevention campaigns be supported with the same kind of robust policy interventions.

### 5.1. What Is Missing?

MP pollution is a relatively new concept, as it was only coined in 2004 [1], and the first reported UN-led action on MPs happened as recently as 2014 [55]. Compared to this short history, the enthusiasm displayed in the global arena on combatting MP pollution, especially in the most recent few years, is encouraging. In particular, the EU targets set to be achieved by 2030 [56] and the current efforts of the UNEA to establish a legally binding framework [56] are commendable. Is this comprehensive enough as an action plan for preventing MP pollution? The answer to this question depends on whether we have a solid understanding of the pathways of MP pollution and whether we trust current waste management practices to prevent any further spread of MPs. Based on the facts/figures discussed here, it is safe to suggest that it is not entirely the case.

Almost all major initiatives campaigning for MP pollution prevention are regurgitating the catchy 3R slogan (reduce, reuse, recycle) followed by proper disposal of the rest of the waste. Of course, 3R combined with proper disposal is a good starting point, but it will not end the issue. In addition, “reduce” seems to be too much to ask at least for now, as plastics have become an irreplaceable material in our lives. Reuse and recycle can be the best recipe for many other types of waste streams; however, when it comes to the plastics this simply means that we are retaining more of the same substances which caused the MP issue in the first place. While incineration and landfilling are highly regarded final waste disposal methods under usual circumstances, as pointed out in the previous sections these solutions are not able to safely contain MPs.

For example, the recent Draft National Strategy to Prevent Plastic Pollution released by the USEPA [47] argues for minimizing plastic pollution through three goals; Goal A mainly involves reduction, Goal B involves proper plastic waste management, and Goal C involves preventing plastic waste in waterways. The underlining assumption of Goals B and C is that “you just have to catch it; the waste management system will take care of the rest,” which seems to be an unfair or incorrect assumption. Another example comes from the washing machine industry. Laundering alone is responsible for releasing half a million tonnes of plastic microfibers each year [66]. When this became an eye-opening topic in the recent years, the washing machine industry rushed to develop MP screens. Nowadays, most modern washing machines are equipped with such filters, which may be able to catch a reasonably high percentage of MP fibers. This is certainly a good trend, and private industry must be commended for the effort. However, the untold part of this story is about what happens to the microfibers caught by the machine; when the filter is full, it needs to be emptied into the domestic trash bin, which is then taken up by the waste management system, which offers plenty of escape routes for the MPs to return to the environment.

### 5.2. How Can These Gaps Be Filled?

It is quite clear that the proper disposal of the currently mismanaged 22% of plastic waste (2019 data) must be a priority. Because this mismanaged portion has already received sufficient attention from ongoing MP prevention campaigns, the following discussion is devoted mainly to arguing “what else” should be done to shut off MP escape routes. Following the MP pathways illustrated in Figure 2, there are a few immediate actions that can/should be taken through appropriate policy interventions.

1. **Obtaining Additional Data.** “What is not measured, can never be managed or improved” is a line popular in all kinds of natural and social science disciplines. This is quite true about MP pollution as well. Enhancement of plastic waste management activities cannot guarantee the desired results until all MP escapes routes within waste management are shut. For this, the escape pathways and relative proportions of MP flows through these routes need to be researched, investigated, and estimated. This will allow for immediate action through diverting funds and paying attention to blocking the most damaging pathways.
2. **Closing the MP cycle between landfills and WWTPs.** Arguably, this is one aspect that is already causing severe damage. The cyclical movement of MPs between landfills and WWTPs provides sufficient time for larger MPs to produce finer secondary MPs that are ultimately released by WWTPs (Figure 2). As discussed, even advanced WWTPs are not able to retain the finest 10% of the MPs, which continue to flow out to the environment together with treated wastewater. Policy interventions are needed to mandate and equip WWTPs to retain as much MP pollution as possible as well as to guarantee safe final disposal of captured MPs.
3. **Enforcing quality control measures for recovery/upcycling associated with plastics.** The above discussion illustrates examples of MP escape through recovery such as land application of biosolids, organic waste composting, and upcycling of bottom ash from incineration. These examples represent sustainable recovery methods. Therefore, any MP pollution preventive actions should be designed in such a way as to not discourage sustainable practices. On the other hand, any new policy interventions can be built on the idea of “plastic-free recovery.” As an example, plastic pollution is certainly unwelcomed by the composting industry and users alike (Figure 3). Enforcing stricter quality control measures to weed out MPs will be beneficial for the composting industry. Similarly, unburned plastics in bottom ash reflects a certain inefficiency associated with the incineration process that could be resolved through stricter environmental laws.
4. **Disposal of unattended plastic items.** As illustrated in Figure 2, a certain fraction of plastic items is neither in use nor labelled as waste. These may be old/abandoned plastic items stacked in garages, barns, backyards, warehouses, or offices (Figure 4). These unattended plastics are not contributing to any meaningful activities in the economy or society, while continuing to release MPs into the environment. It is not easy to estimate the relative size of this fraction. However, observing such items pretty much everywhere in almost all communities, the total volume of unattended plastics could be sufficiently large enough to receive attention. Although it may be difficult to impose a national-level ban on “hoarding” of old/unattended plastics, effective interventions could be made through local/regional policies such as city ordinances. One good example to learn from is how countries have eradicated mosquito-borne diseases by introducing laws/campaigns to remove any potential mosquito breeding grounds from peoples’ backyards, such as unattended cups, cans, buckets, old tires, etc. [67,68].

In closing, it is important to state that the MP pathways/loopholes in the current waste management practices and potential preventive measures pointed out in this discussion are not intended to constitute an exhaustive list; they are just examples. However, we believe that these examples may be helpful in initiating a dialogue to strengthen MP pollution preventive measures through meaningful policy actions. The current literature on the topic provides a clear indication of the increasing global-level enthusiasm on this topic. The legally binding MP pollution prevention treaty that the UNEA is currently negotiating perhaps signals the culmination of these attempts. It would be great if this binding agreement focused on policy interventions targeting the loopholes in current waste management practices related to MPs.

## 6. Conclusions

MP pollution is a growing concern due to the widespread presence of MPs in the environment and their negative effects on human health. MPs are continuously generated and dispersed in the ecosystem by the mismanaged plastic waste as well as due to inefficiencies in the current waste management practices. In addition, millions of tonnes of plastics that are in use or are idle or unattended continue to shed MPs. Combatting MP pollution requires remedial as well as preventive measures. While MP remediation may take time to realize, prevention is something that can happen immediately. However, in order for preventive measures to be effective it is crucial to understand MP escape routes, especially those within existing waste management systems. This may be best addressed with the help of policy interventions. In particular, to make MP preventive measures more effective, policy interventions could help to achieve the following:

- Obtaining additional data needed for prevention planning.
- Closing the MP loop between landfills and WWTPs.
- Enforcing quality control measures on recovery/upcycling associated with plastics.
- Proper disposal of unattended plastics items.

## Figures and Tables

**Table 1:** Fate of plastic waste produced in 2019. Note: data from the Organization for Economic Co-operation and Development OECD [61]; each column total is off from 100% by +/−1%, possibly due to rounding.

What Happened	World Average (%)	OECD Average (%)	Non-OECD Average (%)
Recycling	9	9	10
Incineration	19	29	10
Landfilled	49	57	42
Mismanaged	22	6	39

## Data Availability

Not applicable.
